# Sugarcane cultivar response to glyphosate and trinexapac-ethyl ripeners in Louisiana

**DOI:** 10.1371/journal.pone.0218656

**Published:** 2019-06-20

**Authors:** Douglas J. Spaunhorst, James R. Todd, Anna L. Hale

**Affiliations:** United States Department of Agriculture, Agricultural Research Service, Sugarcane Research Unit, Houma, Louisiana, United States of America; Texas A&M University, UNITED STATES

## Abstract

Sugarcane ripening in Louisiana is necessary to ensure adequate sucrose levels in early-season harvested sugarcane. The response of nine sugarcane cultivar’s yield components to glyphosate and trinexapac-ethyl ripeners was determined in field trials. Glyphosate (210 g ae ha^-1^) and trinexapac-ethyl (200 g ai ha^-1^) treatments failed to increase sucrose yields more than non-ripened sugarcane. Sugarcane ripened with glyphosate or trinexapac-ethyl increased theoretical recoverable sucrose (TRS) 4 to 12% more than non-ripened sugarcane in seven out of nine cultivars, but greater TRS values were counterpoised by lower sugarcane stalk weight. An unintentional consequence of reduced late-season vegetative growth may benefit growers by allowing them to harvest more sugarcane hectares to meet their daily load quota and exposes fewer hectares to a freeze event. The cultivars HoCP 00–950, Ho 09–804, and HoCP 09–840 were not responsive to glyphosate or trinexapac-ethyl ripeners and should not be treated. A delayed harvest from 28 to 49 days after treatment (DAT) coincided with greater TRS values and 17% more sucrose yield.

## Introduction

In Louisiana, sugarcane (*Saccharum* spp.) is cultivated on elevated beds as a perennial crop. A cycle of sugarcane consists of at least three harvests at intervals of 15, 27, and 39 months after planting. Each winter the crop is synchronized by cool temperatures. Typically, the Louisiana sugarcane crop is harvested within a three month window from October to December or early-January. The short harvest season results from potentially freezing temperatures that can occur before the cane crop is completely harvested. Temperatures low enough to compromise yield can occur as early as November through January. A severe to moderate freeze event reduced sucrose true purity by 52% when harvest was delayed by 26 days [1]. In addition to lost sucrose, a freeze event can result in sucrose degradation products (glucose, fructose, dextran, and acids) which can reduce sucrose recovery [1].

Sugarcane growers are compensated on the quantity of sucrose produced per hectare and is the product of theoretical recoverable sucrose (TRS) and cane biomass. Sugarcane cultivars that harbor traits for increased TRS and reduced fiber are desired by growers and millers. Fiber content in most commercial sugarcane cultivars does not exceed 140 g kg^-1^ [2]. Cultivars that produce more fiber increase transportation costs and reduce mill efficiency. Sugarcane milling efficiency is critical due to a limited number of milling facilities available to process the cane crop before a potentially killing frost. In 2017, eleven Louisiana mills processed the state’s 13.6 million Mg cane crop that was produced on 177,000 ha [3].

Unfortunately, TRS early in the harvest season is low because the plant has not fully matured. Environmental factors have shown to influence sugarcane ripening and in sub-tropical cane growing regions sunlight was reported as the most important climatic factor that influenced sugarcane ripening [4]. A common practice to hasten sugarcane maturity is to aerially treat ratoon sugarcane with glyphosate and harvest 28 to 56 days later [5]. Glyphosate and trinexapac-ethyl are two active ingredients that have shown to increase HoCP 96–540 TRS early in the harvest season [6]. Previous research has investigated the effectiveness of the sugarcane ripener glyphosate on cultivars CP 70–321, CP 65–357, and HoCP 96–540. At one time, these cultivars were planted to the majority of Louisiana sugarcane hectares [6–8]. One commercial sugarcane cultivar, on average, is released each year by the Louisiana State University and USDA-ARS Sugarcane Research Unit breeding programs. The response of newly released sugarcane cultivars to ripeners is needed to advise growers on the proper ripener selection. Therefore, it is necessary to continue screening potential commercial and commercially released sugarcane cultivars response to ripeners in Louisiana, particularly glyphosate. The goal of this research was to evaluate the effectiveness of glyphosate and trinexapac-ethyl on sugarcane yield parameters for nine commercial Louisiana sugarcane cultivars and to determine the optimal time interval between application and harvest for maximum sucrose yield.

## Materials and methods

### Site description and experimental design

A field study was conducted in Schriever, LA (29.6372, -90.8395) during the summers of 2016 and 2017 at the USDA-ARS Sugarcane Research Unit Ardoyne Farm, on a Cancienne silt loam (fine-silty, mixed, superactive, nonacidic, thermic Fluvaquentic Endoquepts) to determine the effect of glyphosate and trinexapac-ethyl ripeners on commercial sugarcane cultivars sugar yield components. No specific permissions were required to conduct the field research because the premises were owned by the Federal government and the study did not involve endangered or protected species. The experimental design was a split-plot factorial arrangement of ripener treatments and cultivars with four replications. Whole-plots were ripener treatment with three levels and subplots were nine sugarcane cultivars HoCP 96–540 [9], L 99–226 [10], HoCP 00–950 [11], L 01–283 [12], L 01–299 [13], HoCP 04–838 [14], Ho 07–613, Ho 09–804 [15], and HoCP 09–840 that were randomly allocated to subplots within each whole plot. Glyphosate (Roundup WeatherMax, Monsanto, St. Louis, MO) and trinexapac-ethyl (Moddus, Syngenta Crop Protection, Greensboro, NC) were applied at a rate of 210 g ae ha^-1^ and 200 g ai ha^-1^, respectively. A non-treated control was included for each cultivar for comparison. Plots measured 1.8-m wide and 9.1-m long. A 1-m alley was placed between individual plots to separate sugarcane cultivars.

All cultivars were whole-stalk hand planted with a 10% overlap in raised beds on August 8, 2013 and August 29, 2014 and covered with approximately 7-cm of packed soil. During each cropping cycle the industry standard fertility and weed management program was implemented. In the fall of each year the plant-cane and first ratoon crop were machine harvested. The ripener study was initiated in 2016 and 2017 prior to harvest of the second ratoon crop. Ripener treatments were broadcast applied on August 23, 2016 and August 24, 2017 over the top of erect sugarcane using a 2.7-m wide hand-held CO_2_-pressurized spray boom equipped with nine nozzles and XR8001 flat-fan nozzle tips calibrated to deliver 94 L ha^-1^ at 136 kPa at a speed of 4.8 kph. Two sugarcane cultivars (3.6-m of row width) were treated in a single pass by walking in the wheel furrow between two raised beds. Monthly rainfall totals and monthly average maximum and minimum air temperatures are presented in (Table 1).

**Table 1:** Monthly rainfall (mm) and average monthly maximum and minimum air temperatures (C) at Schriever, Louisiana from January 2016 through December 2017 in comparison to the 30-yr average.

	Rainfall			Air temperature^a^		
Month	2016	2017	30-yr average	2016	2017	30-yr average
	-----------mm-----------				-----------C-----------		
January	81.8	113.0	126.0	19.4 / 5.5	22.2 / 0.0	17.2 / 6.6
February	63.5	48.0	120.9	22.2 / 5.5	23.3 / 9.4	18.8 / 8.3
March	129.0	68.3	121.9	23.3 / 9.4	23.8 / 10.0	22.2 / 11.6
April	238.8	94.0	90.9	25.5 / 14.4	26.6 / 15.5	25.5 / 15.0
May	100.6	332.0	108.9	26.6 / 17.7	28.3 / 18.8	29.4 / 20.0
June	670.8	285.6	185.9	28.8 / 24.4	28.8 / 23.3	31.6 / 22.7
July	208.3	161.8	199.8	28.8 / 25.5	30.0 / 25.5	32.7 / 23.3
August	428.0	388.6	186.9	28.8 / 23.3	28.8 / 24.4	32.7 / 23.3
September	159.5	93.5	143.0	29.4 / 21.1	27.2 / 22.2	31.1 / 21.1
October	0.8	299.7	97.0	25.5 / 16.6	27.7 / 11.1	27.2 / 16.1
November	77.7	7.1	92.9	24.4 / 10.0	23.8 / 10.0	28.3 / 11.1
December	129.5	145.0	104.9	23.3 / 5.5	22.2 / 2.7	18.8 / 7.2

^a^ Maximum / minimum average monthly air temperature and rainfall data were collected from a weather station located near Schriever, LA.

### Data collection

The current recommendation for harvesting ripened sugarcane in Louisiana ranges from 28 to 49 days after treatment (DAT) [16–18]. At four separate timings (28, 35, 42, and 49 DAT) 10 sugarcane stalks were randomly harvested from each plot [18]. Individual stalks were removed approximately 2.5-cm above the soil surface with a machete, stripped of leaves, topped at the last fully expanded internode, and bundled together with twine. A barcode containing sample information was attached to each bundle for tracking purposes and bundles were transported to the laboratory. Within the same day, each bundle of stalks was measured from the base of the plant to the last fully expanded internode to determine plant height and weighed. Whole stalks were crushed using a three-roller mill to extract juice. The extracted juice was analyzed for Brix (percentage by weight of soluble solids) and pol (percentage of apparent sucrose by weight) using a refractometer and saccharimeter, respectively. Methods to calculate TRS (kg Mg^-1^) levels were derived from our colleagues [19]. Millable sugarcane stalks in each plot were counted prior to ripener treatment application. The product of stalk number ha^-1^ and stalk weight (kg stalk^-1^) was used to estimate cane yield (Mg ha^-1^). Sucrose yield (kg ha^-1^) was estimated by multiplying cane yield and TRS. The remaining stand of sugarcane was machine harvested with a Cameco 3500 (John Deere Thibodaux, Thibodaux, LA) single-row chopper harvester after the final hand-cut harvest.

### Statistical analysis

Data were subjected to analysis of variance using PROC MIXED in SAS version 9.2 (SAS Institute Inc., Cary, NC). Ripener treatments and sugarcane cultivar were analyzed as fixed effects, while replication and year were considered random effects. Harvest date was included as a repeated effect in the model. Data were checked for constant variance and normality using PROC UNIVARIATE in SAS, and data were transformed if necessary. Sugarcane stalk weight data were log-transformed and sucrose yield data from hand-cut samples were square root transformed. When data were transformed, mean separation was based on the transformed data but back-transformed means are presented. Treatment means were separated using Tukey’s HSD at the 0.05 level of significance. Sucrose yield data showed no significant interactions among main effects and main effects are discussed separately.

## Results

A ripener treatment by harvest timing (P = 0.0108) interaction influenced sugarcane height. Glyphosate or trinexapac-ethyl ripened sugarcane were 3 to 7% shorter than non-ripened sugarcane at every harvest timing (Table 2). However, trinexapac-ethyl ripened sugarcane were 3% shorter than glyphosate ripened sugarcane at 35 DAT. This result suggested trinexapac-ethyl postponed vegetative growth for at least 35 DAT, but growth resumed and heights were similar to glyphosate-ripened sugarcane at 42 and 49 DAT. Sugarcane height increased 5 to 8% when harvest was delayed one or two weeks after the 35 DAT harvest timing for trinexapac-ethyl and non-ripened sugarcane (Table 2). A week by week increase in sugarcane height from 28 to 49 DAT was not observed with glyphosate-ripened sugarcane. However, glyphosate-ripened sugarcane heights increased by 4% when harvest was delayed by two weeks, 28 to 42 DAT or 35 to 49 DAT (Table 2). Richard [20] showed sub-lethal glyphosate rates (0.1 to 0.2 kg ha^-1^) temporarily delayed sugarcane stalk elongation when treated at the grand growth phase. There was a significant cultivar by ripener treatment interaction for sugarcane height (P = 0.0119). Both glyphosate and trinexapac-ethyl reduced plant heights of L 99–226, L 01–299, and Ho 07–613 by 8% when compared to non-ripened L 99–226, L 01–299, and Ho 07–613 (Table 3). However, neither of the ripener treatment reduced HoCP 96–540, Ho 09–804, and HoCP 09–840 heights. This result suggested that the aforementioned cultivars were potentially more tolerant to reduced rates of glyphosate than L 99–226, L 01–299, and Ho 07–613. Height data also revealed that some cultivars were more sensitive to trinexapac-ethyl than glyphosate. Trinexapac-ethyl ripened HoCP 00–950 and HoCP 04–838 measured 2 and 3% less than glyphosate-ripened HoCP 00–950 and HoCP 04–838, respectively.

**Table 2:** Effect of sugarcane cultivar and ripener treatment on sugarcane height at 28, 35, 42, and 49 days after treatment (DAT) in 2016 and 2017 at Schriever, LA.

	Sugarcane height^a^^b^							
Factor	28 DAT		35 DAT		42 DAT		49 DAT	
	---------------------------------cm---------------------------------							
Cultivar								
HoCP 96–540	212 bc	A	214 bc	A	221 bc	A	224 bc	A
L 99–226	217 ab	B	227 a	A	235 a	A	238 a	A
HoCP 00–950	186 e	B	189 e	B	200 d	AB	206 d	A
L 01–283	214 abc	B	220 ab	AB	225 ab	AB	232 ab	A
L 01–299	226 a	A	229 a	A	231 ab	A	238 a	A
HoCP 04–838	196 de	A	191 e	A	201 d	A	207 d	A
Ho 07–613	214 bc	B	223 ab	AB	222 bc	AB	229 ab	A
Ho 09–804	204 cd	B	204 cd	B	219 bc	A	225 bc	A
HoCP 09–840	195 de	C	199 de	BC	211 cd	AB	215 cd	A
Treatment								
Nontreated	213 a	B	217 a	B	228 a	A	235 a	A
Glyphosate	206 b	C	210 b	BC	214 b	AB	218 b	A
Trinexapac-ethyl	202 b	B	204 c	B	213 b	A	218 b	A

^a^ Sugarcane stalks were measured from the base of the plant to the last fully expanded internode.

^b^ Means within cultivar and treatment that are followed by the same lower case letter and means within DAT that are followed by the same upper case letter are not significantly different according to Tukey HSD (0.05).

**Table 3:** Sugarcane heights as affected by sugarcane cultivar and ripener treatment in 2016 and 2017 at Schriever, LA.

	Sugarcane height^a^^b^					
	Nontreated		Glyphosate		Trinexapac-ethyl	
	---------------------------------cm---------------------------------					
Cultivar						
HoCP 96–540	225 cd	A	213 abc	A	215 ab	A
L 99–226	242 ab	A	223 ab	B	223 a	B
HoCP 00–950	202 f	A	193 d	A	190 e	B
L 01–283	230 bcd	A	223 a	AB	215 ab	B
L 01–299	244 a	A	224 a	B	224 a	B
HoCP 04–838	205 f	A	198 d	A	193 de	B
Ho 07–613	232 abc	A	219 ab	B	213 abc	B
Ho 09–804	218 de	A	211 bc	A	209 bc	A
HoCP 09–840	208 ef	A	205 cd	A	203 cd	A

^a^ Sugarcane stalks were measured from the base of the plant to the last fully expanded internode.

^b^ Means within a ripener that are followed by the same lower case letter and means within a cultivar that are followed by the same upper case letter are not significantly different according to Tukey HSD (0.05).

Sugarcane stalk weights were variable between cultivar and did not increase from 28 to 49 DAT (Table 4). A similar trend for ripener treatment also showed no increase in sugarcane stalk weight when harvested from 28 to 49 DAT. Non-ripened sugarcane stalk weight was 8 to 13% greater than glyphosate- or trinexapac-ethyl-ripened sugarcane at 28, 42, and 49 DAT. Greater stalk weight for non-ripened sugarcane when compared to ripened sugarcane at 28, 42, and 49 DAT likely resulted from continued plant growth (Table 4). A common characteristic of trinexapac-ethyl and glyphosate treated sugarcane when compared to non-ripened sugarcane is shortening of internodes and cessation of apical growth, respectively [18,21].

**Table 4:** Effect of sugarcane cultivar and ripener treatments on sugarcane stalk fresh weight from whole stalk samples at 28, 35, 42, and 49 days after treatment (DAT) in 2016 and 2017 at Schriever, LA.

	Sugarcane stalk weight^a^							
Factor	28 DAT		35 DAT		42 DAT		49 DAT	
	---------------------------------kg stalk^-1^---------------------------------							
Cultivar								
HoCP 96–540	1.53 bc	A	1.58 bc	A	1.49 bcd	A	1.50 c	A
L 99–226	1.84 a	A	1.84 a	A	1.95 a	A	1.99 a	A
HoCP 00–950	1.50 bc	A	1.48 cd	A	1.58 bc	A	1.52 c	A
L 01–283	1.36 cde	A	1.42 cde	A	1.44 cd	A	1.46 cd	A
L 01–299	1.47 bcd	A	1.50 cd	A	1.41 cd	A	1.44 cd	A
HoCP 04–838	1.47 bcd	A	1.37 cde	A	1.36 cd	A	1.43 cd	A
Ho 07–613	1.64 ab	A	1.74 ab	A	1.73 ab	A	1.76 b	A
Ho 09–804	1.26 de	A	1.29 de	A	1.37 cd	A	1.35 cd	A
HoCP 09–840	1.23 e	A	1.22 e	A	1.28 d	A	1.28 d	A
Treatment								
Nontreated	1.55 a	A	1.53 a	A	1.62 a	A	1.60 a	A
Glyphosate	1.44 b	A	1.50 a	A	1.43 b	A	1.48 b	A
Trinexapac-ethyl	1.44 b	A	1.45 a	A	1.50 b	A	1.49 b	A

^a^ Means within a DAT that are followed by the same lower case letter and means within a cultivar and treatment that are followed by the same upper case letter are not significantly different according to Tukey HSD (0.05). Mean separation is from the log transformation, presented means are from back-transformed data.

A cultivar by harvest timing interaction influenced TRS (P = 0.0018). The TRS level for every cultivar increased when harvest was delayed from 28 to 49 DAT (Table 5). The only cultivar to increase TRS at each harvest timing was HoCP 96–540. TRS was maximized for HoCP 04–838, Ho 07–613, Ho 09–804, and HoCP 09–840 when plants were harvested no earlier than 42 DAT. This result suggested HoCP 04–838, Ho 07–613, Ho 09–804, and HoCP 09–840 were early-maturing cultivars. However, maximum TRS was achieved at 49 DAT with HoCP 96–540, L 99–226, and L 01–299. The cultivar by ripener treatment interaction was also significant for TRS (P = 0.0001). TRS values for glyphosate- or trinexapac-ethyl-treated HoCP 96–540, L 99–226, L 01–299, HoCP 04–838, and Ho 07–613 were 4 to 12% more than non-ripened cultivars (Table 6). However, ripeners failed to increase TRS for HoCP 00–950, Ho 09–804, and HoCP 09–840 when compared to non-ripened HoCP 00–950, Ho 09–804, and HoCP 09–840.

**Table 5:** Effect of sugarcane cultivar on theoretical recoverable sucrose from whole stalk samples at 28, 35, 42, and 49 days after treatment (DAT) in 2016 and 2017 at Schriever, LA.

	Theoretical recoverable sucrose^a^							
	28 DAT		35 DAT		42 DAT		49 DAT	
	---------------------------------kg Mg^-1^---------------------------------							
Cultivar								
HoCP 96–540	119 e	D	123 e	C	131 d	B	140 e	A
L 99–226	127 cd	C	131 cd	C	140 bc	B	147 cd	A
HoCP 00–950	147 a	B	145 a	B	153 a	AB	158 a	A
L 01–283	143 a	C	144 ab	BC	151 a	AB	154 ab	A
L 01–299	122 de	C	128 de	C	137 cd	B	144 de	A
HoCP 04–838	130 bc	B	134 cd	B	143 bc	A	148 cd	A
Ho 07–613	135 b	B	137 bc	B	146 ab	A	151 bc	A
Ho 09–804	133 bc	B	135 cd	B	142 bc	A	146 cd	A
HoCP 09–840	130 bc	B	132 cd	B	142 bc	A	147 cd	A

^a^ Means within a DAT that are followed by the same lower case letter and means within a cultivar that are followed by the same upper case letter are not significantly different according to Tukey HSD (0.05).

**Table 6:** Theoretical recoverable sucrose as affected by ripener treatment in 2016 and 2017 at Schriever, LA.

	Theoretical recoverable sucrose^a^					
	Nontreated		Glyphosate		Trinexapac-ethyl	
	---------------------------------kg Mg^-1^---------------------------------					
Cultivar						
HoCP 96–540	122 e	B	134 d	A	129 e	A
L 99–226	129 cd	C	144 bc	A	136 cd	B
HoCP 00–950	148 a	A	152 a	A	152 a	A
L 01–283	145 a	B	147 ab	AB	151 a	A
L 01–299	127 de	B	139 cd	A	134 de	A
HoCP 04–838	133 bc	B	144 bc	A	139 bcd	A
Ho 07–613	135 b	B	147 ab	A	144 b	A
Ho 09–804	138 b	A	139 cd	A	140 bc	A
HoCP 09–840	135 b	A	139 cd	A	139 bcd	A

^a^ Means within a ripener treatment that are followed by the same lower case letter and means within a cultivar that are followed by the same upper case letter are not significantly different according to Tukey HSD (0.05).

Sucrose yield is determined by TRS and sugarcane yield. It is important to note that the 2017 state average sucrose yield was 9,869 kg ha^-1^ [3]. An average sucrose yield of that magnitude had never been achieved in Louisiana’s history and likely resulted from favorable growing conditions and dry harvest conditions that favored sucrose recovery [22]. In the current study, glyphosate and trinexapac-ethyl ripeners failed to increase sucrose yield (Table 7). In a different study, Orgeron et al. [6] reported 210 g ha^-1^ of glyphosate and 350 g ha^-1^ of trinexapac-ethyl applied separately failed to increase HoCP 96–540 sucrose yield. Sucrose yield was sensitive to harvest timing and increased by 17% when harvest was delayed from 28 to 49 DAT (Table 7).

**Table 7:** Effect of sugarcane cultivar and ripener treatments on sucrose yield from whole stalk samples at 28, 35, 42, and 49 days after treatment (DAT) in 2016 and 2017 at Schriever, LA.

Factor	Sucrose yield^a^^b^
	kg ha^-1^
Cultivar	
HoCP 96–540	10,402 cd
L 99–226	10,570 cd
HoCP 00–950	10,211 cd
L 01–283	11,533 b
L 01–299	10,467 cd
HoCP 04–838	10,035 d
Ho 07–613	12,482 a
Ho 09–804	10,844 bc
HoCP 09–840	10,350 cd
Treatment	
Nontreated	10,967 a
Glyphosate	10,652 a
Trinexapac-ethyl	10,668 a
Days after ripener treatment	
28	9,954 c
35	10,366 c
42	11,129 b
49	11,616 a

^a^ Data were square root transformed and means were back transformed for presentation.

^b^ Means within cultivar, treatment, and days after ripener treatment that are followed by the same lower case letter are not significantly different according to Tukey HSD (0.05).

## Discussion

The current data showed TRS increased for all cultivars when harvest was delayed to 49 DAT and was likely the result of natural plant ripening stimulated by lower air temperature as the harvest season progressed (Table 1). Many studies have shown cooler air temperatures promoted sugarcane ripening [23,24]. Higher than normal annual rainfall was recorded during both years the study was conducted, especially in June and August (Table 1). However, the majority of August rainfall occurred before ripeners were applied. Previous research showed the effect of rainfall on cultivar ripening was marginally correlated (-0.191 to 0.174) and unlikely influenced ripener treatments in the current study [4]. Water-logged soil conditions have resulted in reduced sucrose and sugarcane yield [25]. In the present study, prolonged saturated soil conditions were not observed as the studies were conducted on graded (0.2%) silt loam soils that drained down gradient.

A desired outcome from glyphosate or trinexapac-ethyl ripened sugarcane was to produce more sucrose yield than non-ripened sugarcane. Data from this study showed no increase in sucrose yield for glyphosate or trinexapac-ethyl ripened sugarcane when compared to non-ripened sugarcane. Some research showed mixed results for increased sucrose yield from glyphosate ripened sugarcane when compared to non-ripened sugarcane [26]. In the previously mentioned study, increased sucrose yield likely resulted from LCP 85–384 being highly responsive to glyphosate [26]. Our colleagues showed glyphosate ripened HoCP 96–540 increased TRS, but the reduction in sugarcane biomass offset the gain in TRS and sucrose yield was similar to nonripened sugarcane [27]. Trinexapac-ethyl treated sugarcane has shown to reduce internode elongation and resulted in shorter plants [28]. Interestingly, the average monthly maximum air temperatures in 2016 and 2017 from June to September were 2 to 4 C cooler than the 30-yr average. Research has shown the relationship between incident sunlight and temperature on sugarcane ripening are closely associated and may have contributed to the lack of difference in sucrose yield between ripener treatments in the present study [4]. The greatest gain in TRS from a ripener application applied in a sub-tropical environment was reported to occur when sugarcane was ripened earlier in the harvest season when compared to sugarcane ripened later in the harvest season [29]. In the current study, the ripener schedule was aimed for sugarcane harvest to begin late-September (28 DAT harvest) and finish mid-October (49 DAT harvest) to achieve maximum TRS potential from the ripener treatment.

Most Louisiana sugarcane mills compensate growers for the glyphosate ripener costs, but not trinexapac-ethyl. Both the mill and grower benefit from the glyphosate ripener treatment because less cane biomass is harvested, transported from field to mill, and processed through the mill. This allows growers to harvest more hectares of sugarcane to fill their daily quota and complete harvest before a killing frost occurs. The reduction in plant height and stalk weight from the glyphosate ripener was counterpoised by increased TRS levels for glyphosate treated sugarcane, especially for cultivars L 99–226, L 01–299, and Ho 07–613. Interestingly for cultivars HoCP 96–540 and HoCP 04–838, the glyphosate ripener did not reduce plant height but increased TRS. In greenhouse conditions, glyphosate applied at 36 g ha^-1^ to SP 80–1842 increased biomass 25% more than the non-treated [30]. Other researchers reported sugarcane biomass increased nearly 30% more than the non-treated when exposed to 18 g ha^-1^ of glyphosate at 60 DAT, and the hormetic effect was greater at 60 d when compared to 40 d [31]. The labeled ripener rate for glyphosate and trinexapac-ethyl ranged from 158 to 474 g ha^-1^ and 200 to 346 g ha^-1^, respectively. The application to harvest interval for glyphosate and trinexapac-ethyl ripened sugarcane in Louisiana should not exceed 42 and 60 DAT, respectively. Although the current study showed no loss in sucrose yield when glyphosate ripened sugarcane was harvested at 49 DAT, but glyphosate ripened sugarcane harvested at 60 DAT has shown to reduce crop biomass by 4.9 Mg ha^-1^ and sucrose yield by 900 kg ha^-1^ when compared with sugarcane harvested at 40 DAT [26].

Screening compounds that result in early-season sugarcane maturation has the potential to greatly impact the Louisiana industry, as growers race to harvest their entire crop before a potential freeze event occurs [18]. Although this study showed nine commercial sugarcane cultivars do not respond by increasing sucrose yield when treated to glyphosate and trinexapac-ethyl ripeners, application of glyphosate to ripen sugarcane will likely continue for reasons previously mentioned. Annual glyphosate ripener applications made during a three-year crop cycle can reduce sugarcane spring regrowth [7,32]. Reduced crop vigor may be limited to sugarcane produced in sub-tropical environments or under stressed conditions [33]. Trinexapac-ethyl can be applied to all crops in the sugarcane cycle. However, glyphosate is limited to ratoon sugarcane crops in Louisiana and the final ratoon in Florida [5,18]. Caution should be taken to avoid treating stressed sugarcane and cultivars that do not respond with glyphosate due to potential negative effects on subsequent ratoons.

## Supporting information

S1 Dataset Sugarcane height, stalk weight, theoretical recoverable sucrose, and sucrose yield at 28, 35, 42 and 49 days after treatment to glyphosate (210 g ae ha^-1^) and trinexapac-ethyl (200 g ai ha^-1^) applied separately. (PDF)
