# Supplementary material for: Sugarcane cultivar response to glyphosate and trinexapac-ethyl ripeners in Louisiana
Source: PLoS One. 2019 Jun 20;14(6):e0218656. doi: 10.1371/journal.pone.0218656 (PMC6586340; doi:10.1371/journal.pone.0218656)
Supplement: S1 Dataset — (PDF) [file pone.0218656.s001.pdf]

S1 Dataset. Sugarcane height, stalk weight, theoretical recoverable sucrose, and sucrose yield at 28, 35, 42 and 49 days after treatment to glyphosate (210 g ae ha-1) and trinexapac-ethyl (200 g ai ha-1) applied separately.

variety	trt	rep	year	ratingdate	stalkheight	stalkweight	trs	sucrose yield
840	moddus	1	16	28	175	0.98	134	7434
840	moddus	2	16	28	178	1	138	7910
840	moddus	3	16	28	180	0.94	120	6619
840	moddus	4	16	28	188	1.06	140	8298
840	control	1	16	28	196	1.24	124	8787
840	control	2	16	28	188	1.05	130	7327
840	control	3	16	28	188	1.24	134	9594
840	control	4	16	28	188	1.08	119	7333
840	glyphosate	1	16	28	188	0.98	140	7280
840	glyphosate	2	16	28	165	0.81	130	5591
840	glyphosate	3	16	28	178	0.95	130	6916
840	glyphosate	4	16	28	198	1.29	137	.
804	moddus	1	16	28	185	0.96	143	8061
804	moddus	2	16	28	185	1.11	148	8885
804	moddus	3	16	28	173	1.02	137	8770
804	moddus	4	16	28	170	0.94	124	6673
804	control	1	16	28	180	0.92	132	6005
804	control	2	16	28	180	0.8	132	5149
804	control	3	16	28	196	1.32	130	9181
804	control	4	16	28	193	1.07	129	8198
804	glyphosate	1	16	28	188	1.1	141	8177
804	glyphosate	2	16	28	191	1.05	148	6908
804	glyphosate	3	16	28	208	1.28	142	10228
804	glyphosate	4	16	28	201	1.13	133	9605
950	moddus	1	16	28	175	1.32	153	7380
950	moddus	2	16	28	173	1.37	155	8915
950	moddus	3	16	28	168	1.29	142	5885
950	moddus	4	16	28	188	1.46	154	10141
950	control	1	16	28	185	1.32	139	6634
950	control	2	16	28	185	1.09	153	6793
950	control	3	16	28	180	1.37	133	6741
950	control	4	16	28	193	1.35	141	7581
950	glyphosate	1	16	28	180	1.39	155	8516
950	glyphosate	2	16	28	168	0.97	146	5853
950	glyphosate	3	16	28	168	1.24	145	8472
950	glyphosate	4	16	28	188	1.35	162	7236
299	moddus	1	16	28	211	1.53	135	7461
299	moddus	2	16	28	206	1.22	127	8414
299	moddus	3	16	28	193	1.28	134	7810
299	moddus	4	16	28	208	1.18	123	7232
299	control	1	16	28	216	1.42	130	7551
299	control	2	16	28	226	1.56	103	8154
299	control	3	16	28	211	1.29	113	7748

299	control	4	16	28	231	1.46	108	7142
299	glyphosate	1	16	28	178	1.15	156	4651
299	glyphosate	2	16	28	206	1.27	139	7488
299	glyphosate	3	16	28	221	1.44	129	8192
299	glyphosate	4	16	28	213	1.28	122	6919
838	moddus	1	16	28	163	1.08	136	6332
838	moddus	2	16	28	180	1.13	132	7623
838	moddus	3	16	28	198	1.26	129	9138
838	moddus	4	16	28	185	1.1	137	8529
838	control	1	16	28	173	1.13	126	5593
838	control	2	16	28	188	1.28	130	6541
838	control	3	16	28	213	1.51	116	7891
838	control	4	16	28	188	1.5	111	7667
838	glyphosate	1	16	28	152	1.01	143	5004
838	glyphosate	2	16	28	163	1.04	151	6823
838	glyphosate	3	16	28	191	1.22	141	8731
838	glyphosate	4	16	28	175	1.07	144	7779
613	moddus	1	16	28	168	1.05	149	5869
613	moddus	2	16	28	191	1.46	136	8949
613	moddus	3	16	28	183	1.3	144	9774
613	moddus	4	16	28	201	1.57	140	11267
613	control	1	16	28	201	1.7	141	9705
613	control	2	16	28	185	1.2	134	7074
613	control	3	16	28	201	1.34	126	8094
613	control	4	16	28	208	1.4	127	9102
613	glyphosate	1	16	28	183	1.24	153	8606
613	glyphosate	2	16	28	188	1.27	143	7828
613	glyphosate	3	16	28	191	1.46	147	10056
613	glyphosate	4	16	28	191	1.18	148	7653
226	moddus	1	16	28	188	1.4	128	4916
226	moddus	2	16	28	198	1.77	130	7883
226	moddus	3	16	28	185	1.35	114	6239
226	moddus	4	16	28	188	1.73	128	9319
226	control	1	16	28	208	1.65	126	6227
226	control	2	16	28	221	1.68	123	6068
226	control	3	16	28	206	1.47	120	7283
226	control	4	16	28	216	1.48	124	6139
226	glyphosate	1	16	28	193	1.43	150	6216
226	glyphosate	2	16	28	208	1.61	148	8705
226	glyphosate	3	16	28	201	1.22	134	5896
226	glyphosate	4	16	28	206	1.82	144	10745
283	moddus	1	16	28	191	1.1	147	6800
283	moddus	2	16	28	213	1.42	150	10621
283	moddus	3	16	28	208	1.22	157	9708
283	moddus	4	16	28	203	1.38	154	10331
283	control	1	16	28	216	1.13	142	6980
283	control	2	16	28	196	1.03	140	7233

283	control	3	16	28	231	1.42	136	7916
283	control	4	16	28	198	1.17	140	6692
283	glyphosate	1	16	28	188	1.21	154	8803
283	glyphosate	2	16	28	208	1.41	129	8844
283	glyphosate	3	16	28	201	0.92	145	7090
283	glyphosate	4	16	28	221	1.48	147	10999
540	moddus	1	16	28	198	1.41	136	9945
540	moddus	2	16	28	168	0.85	119	4223
540	moddus	3	16	28	191	0.94	118	5519
540	moddus	4	16	28	183	1.18	127	7928
540	control	1	16	28	198	1.3	129	8204
540	control	2	16	28	203	1.5	115	8309
540	control	3	16	28	221	1.64	112	9762
540	control	4	16	28	203	1.49	114	7979
540	glyphosate	1	16	28	193	1.33	137	7595
540	glyphosate	2	16	28	191	1.21	129	5236
540	glyphosate	3	16	28	203	1.46	128	8708
540	glyphosate	4	16	28	196	1.13	117	6721
950	moddus	1	17	28	191	1.78	150	12045
950	moddus	2	17	28	185	1.72	150	12250
950	moddus	3	17	28	196	1.8	150	13402
950	moddus	4	17	28	191	1.69	149	13881
950	control	1	17	28	201	1.57	151	11806
950	control	2	17	28	193	1.64	147	13732
950	control	3	17	28	185	1.5	141	8900
950	control	4	17	28	206	1.94	142	13635
950	glyphosate	1	17	28	183	1.45	141	10401
950	glyphosate	2	17	28	203	1.85	141	10987
950	glyphosate	3	17	28	193	1.66	148	11565
950	glyphosate	4	17	28	193	1.86	140	11285
840	moddus	1	17	28	198	1.1	131	8788
840	moddus	2	17	28	218	1.4	132	11235
840	moddus	3	17	28	196	1.43	127	10846
840	moddus	4	17	28	203	1.52	130	13543
840	control	1	17	28	206	1.39	130	10750
840	control	2	17	28	229	1.65	128	14030
840	control	3	17	28	198	1.47	130	12923
840	control	4	17	28	185	1.31	129	10794
840	glyphosate	1	17	28	206	1.35	130	10577
840	glyphosate	2	17	28	231	1.47	118	10671
840	glyphosate	3	17	28	211	1.57	134	13081
840	glyphosate	4	17	28	198	1.19	133	9999
226	moddus	1	17	28	224	1.76	120	9933
226	moddus	2	17	28	239	2.28	127	12299
226	moddus	3	17	28	251	2.12	118	9487
226	moddus	4	17	28	226	2.23	118	10139
226	control	1	17	28	236	2.27	109	12596

226	control	2	17	28	249	2.25	122	13979
226	control	3	17	28	249	2.04	123	10983
226	control	4	17	28	224	2.43	121	12621
226	glyphosate	1	17	28	218	1.61	126	10062
226	glyphosate	2	17	28	224	2.14	136	13356
226	glyphosate	3	17	28	241	2.19	124	13027
226	glyphosate	4	17	28	224	2.35	128	13872
283	moddus	1	17	28	224	1.28	149	10557
283	moddus	2	17	28	231	1.53	144	15028
283	moddus	3	17	28	213	1.31	147	10914
283	moddus	4	17	28	206	1.34	147	11762
283	control	1	17	28	221	1.58	149	13312
283	control	2	17	28	244	1.78	135	14638
283	control	3	17	28	226	1.76	138	16321
283	control	4	17	28	221	1.59	140	13571
283	glyphosate	1	17	28	229	1.51	137	12337
283	glyphosate	2	17	28	229	1.44	136	12315
283	glyphosate	3	17	28	221	1.48	134	13478
283	glyphosate	4	17	28	211	1.2	129	8182
299	moddus	1	17	28	246	1.48	117	10869
299	moddus	2	17	28	234	1.58	121	12191
299	moddus	3	17	28	216	1.55	130	13351
299	moddus	4	17	28	246	1.55	117	11902
299	control	1	17	28	259	1.76	109	12431
299	control	2	17	28	226	1.73	121	12426
299	control	3	17	28	236	1.76	117	13070
299	control	4	17	28	274	1.82	109	12736
299	glyphosate	1	17	28	254	1.65	118	12097
299	glyphosate	2	17	28	259	1.84	118	13049
299	glyphosate	3	17	28	208	1.15	128	9011
299	glyphosate	4	17	28	241	1.41	116	10207
613	moddus	1	17	28	226	1.51	129	10304
613	moddus	2	17	28	213	1.74	135	13266
613	moddus	3	17	28	251	2.21	127	14732
613	moddus	4	17	28	236	1.86	142	15141
613	control	1	17	28	244	1.65	116	10939
613	control	2	17	28	229	1.98	126	15573
613	control	3	17	28	241	1.9	125	13438
613	control	4	17	28	251	2.31	121	15768
613	glyphosate	1	17	28	257	2.45	131	16895
613	glyphosate	2	17	28	206	1.48	137	12080
613	glyphosate	3	17	28	241	2.13	132	13038
613	glyphosate	4	17	28	241	2.05	130	14992
540	moddus	1	17	28	221	1.58	123	10381
540	moddus	2	17	28	218	1.82	120	12937
540	moddus	3	17	28	218	2.1	110	13156
540	moddus	4	17	28	231	1.65	110	9100

540	control	1	17	28	234	1.94	113	12070
540	control	2	17	28	251	1.86	111	12053
540	control	3	17	28	211	1.92	111	13209
540	control	4	17	28	229	1.68	101	9782
540	glyphosate	1	17	28	226	1.53	129	10739
540	glyphosate	2	17	28	239	1.9	129	15656
540	glyphosate	3	17	28	229	1.75	119	13114
540	glyphosate	4	17	28	246	1.66	102	10660
838	moddus	1	17	28	188	1.39	130	8686
838	moddus	2	17	28	213	2.22	133	16784
838	moddus	3	17	28	193	1.37	134	9180
838	moddus	4	17	28	211	1.61	122	10886
838	control	1	17	28	211	1.7	126	11098
838	control	2	17	28	244	2.38	130	.
838	control	3	17	28	208	1.8	123	12746
838	control	4	17	28	203	1.93	113	12407
838	glyphosate	1	17	28	224	1.8	127	12170
838	glyphosate	2	17	28	221	1.52	139	12733
838	glyphosate	3	17	28	193	1.43	137	10383
838	glyphosate	4	17	28	231	1.84	121	13635
804	moddus	1	17	28	229	1.61	126	11823
804	moddus	2	17	28	208	1.23	132	9126
804	moddus	3	17	28	224	1.61	132	13066
804	moddus	4	17	28	229	1.49	132	13874
804	control	1	17	28	224	1.37	135	11309
804	control	2	17	28	221	1.57	135	13317
804	control	3	17	28	226	1.57	125	12414
804	control	4	17	28	224	1.39	126	10126
804	glyphosate	1	17	28	218	1.59	138	14299
804	glyphosate	2	17	28	221	1.61	127	11573
804	glyphosate	3	17	28	216	1.42	126	11990
804	glyphosate	4	17	28	198	1.17	127	8984
840	moddus	1	16	35	175	1.15	129	8366
840	moddus	2	16	35	180	0.92	136	7151
840	moddus	3	16	35	198	1.07	140	8790
840	moddus	4	16	35	188	1.41	137	10827
840	control	1	16	35	188	0.97	116	6408
840	control	2	16	35	185	0.98	130	6853
840	control	3	16	35	201	1.13	121	7855
840	control	4	16	35	196	1.28	127	9297
840	glyphosate	1	16	35	198	1.3	140	9629
840	glyphosate	2	16	35	180	0.87	137	6340
840	glyphosate	3	16	35	201	0.97	138	7521
840	glyphosate	4	16	35	183	1.15	121	7921
804	moddus	1	16	35	193	1.17	125	8626
804	moddus	2	16	35	180	1.03	135	7548
804	moddus	3	16	35	183	0.98	137	8466

804	moddus	4	16	35	191	1.1	147	9212
804	control	1	16	35	203	1.35	140	9336
804	control	2	16	35	201	1.15	135	7548
804	control	3	16	35	191	1.14	136	8338
804	control	4	16	35	201	1.19	129	9117
804	glyphosate	1	16	35	183	1.14	144	8644
804	glyphosate	2	16	35	165	0.76	126	4262
804	glyphosate	3	16	35	178	1.01	132	7498
804	glyphosate	4	16	35	201	1.12	141	10070
950	moddus	1	16	35	152	1.03	144	5423
950	moddus	2	16	35	168	1.13	146	6940
950	moddus	3	16	35	183	1.34	143	6135
950	moddus	4	16	35	170	1.4	148	9363
950	control	1	16	35	173	1.12	142	5737
950	control	2	16	35	178	1.26	147	7537
950	control	3	16	35	193	1.44	138	7352
950	control	4	16	35	193	1.26	138	6933
950	glyphosate	1	16	35	160	1.04	143	5904
950	glyphosate	2	16	35	183	1.19	149	7371
950	glyphosate	3	16	35	175	1.24	151	8798
950	glyphosate	4	16	35	170	1.28	149	6302
299	moddus	1	16	35	206	1.12	142	5734
299	moddus	2	16	35	221	1.09	123	7288
299	moddus	3	16	35	188	1.12	129	6563
299	moddus	4	16	35	213	1.37	137	9386
299	control	1	16	35	221	1.47	124	7456
299	control	2	16	35	213	1.33	126	8521
299	control	3	16	35	216	1.31	123	8513
299	control	4	16	35	218	1.25	113	6426
299	glyphosate	1	16	35	203	1.09	148	4173
299	glyphosate	2	16	35	206	1.26	146	7807
299	glyphosate	3	16	35	193	1.28	135	7602
299	glyphosate	4	16	35	221	1.37	140	8505
838	moddus	1	16	35	165	0.91	127	4985
838	moddus	2	16	35	163	1.02	139	7270
838	moddus	3	16	35	175	1.16	136	8894
838	moddus	4	16	35	185	1.39	137	10788
838	control	1	16	35	168	1.16	121	5512
838	control	2	16	35	180	1.06	126	5226
838	control	3	16	35	206	1.27	119	6854
838	control	4	16	35	198	1.12	122	6301
838	glyphosate	1	16	35	155	0.98	146	4955
838	glyphosate	2	16	35	165	1.08	147	6857
838	glyphosate	3	16	35	180	1.36	147	10126
838	glyphosate	4	16	35	173	1.35	148	10105
613	moddus	1	16	35	168	1.03	140	5386
613	moddus	2	16	35	188	1.3	152	8905

613	moddus	3	16	35	201	1.24	150	9722
613	moddus	4	16	35	198	1.29	136	9006
613	control	1	16	35	201	1.43	141	8163
613	control	2	16	35	206	1.38	136	8266
613	control	3	16	35	224	1.82	127	11136
613	control	4	16	35	224	1.87	130	12406
613	glyphosate	1	16	35	193	1.35	155	9506
613	glyphosate	2	16	35	218	1.85	147	11670
613	glyphosate	3	16	35	208	1.62	158	11993
613	glyphosate	4	16	35	198	1.47	151	9766
226	moddus	1	16	35	196	1.39	129	4932
226	moddus	2	16	35	196	1.55	137	7294
226	moddus	3	16	35	211	1.49	128	7754
226	moddus	4	16	35	193	1.6	134	9025
226	control	1	16	35	224	1.78	133	7104
226	control	2	16	35	201	1.6	111	5224
226	control	3	16	35	229	1.59	116	7661
226	control	4	16	35	244	1.97	121	7966
226	glyphosate	1	16	35	203	1.57	158	7176
226	glyphosate	2	16	35	206	1.68	152	9283
226	glyphosate	3	16	35	208	1.79	129	8365
226	glyphosate	4	16	35	203	1.68	146	10120
283	moddus	1	16	35	196	1.23	146	7569
283	moddus	2	16	35	216	1.49	145	10737
283	moddus	3	16	35	206	1.5	157	11976
283	moddus	4	16	35	173	0.9	154	6749
283	control	1	16	35	206	1.18	140	7185
283	control	2	16	35	236	1.33	137	9151
283	control	3	16	35	211	1.29	132	7026
283	control	4	16	35	198	1.05	139	5965
283	glyphosate	1	16	35	203	1.22	147	8506
283	glyphosate	2	16	35	203	1.08	149	7848
283	glyphosate	3	16	35	196	1.18	147	9177
283	glyphosate	4	16	35	218	1.55	153	11947
540	moddus	1	16	35	188	1.36	135	9480
540	moddus	2	16	35	175	1.18	123	6048
540	moddus	3	16	35	206	1.21	132	7960
540	moddus	4	16	35	193	1.1	116	6767
540	control	1	16	35	201	1.25	118	7192
540	control	2	16	35	183	1.01	116	5621
540	control	3	16	35	226	1.4	118	8815
540	control	4	16	35	208	1.35	104	6635
540	glyphosate	1	16	35	193	1.65	140	9651
540	glyphosate	2	16	35	188	1.32	139	6164
540	glyphosate	3	16	35	203	1.16	131	7063
540	glyphosate	4	16	35	198	1.24	129	8125
950	moddus	1	17	35	188	1.72	142	11038

950	moddus	2	17	35	191	1.6	148	11225
950	moddus	3	17	35	203	1.49	152	11262
950	moddus	4	17	35	201	1.99	151	16580
950	control	1	17	35	193	1.69	150	12562
950	control	2	17	35	211	1.83	151	15713
950	control	3	17	35	206	1.64	141	9749
950	control	4	17	35	196	1.69	137	11463
950	glyphosate	1	17	35	208	1.85	151	14187
950	glyphosate	2	17	35	201	1.35	143	8109
950	glyphosate	3	17	35	208	2.16	146	14870
950	glyphosate	4	17	35	231	1.82	143	11308
840	moddus	1	17	35	196	1.27	127	9783
840	moddus	2	17	35	180	1.14	133	9205
840	moddus	3	17	35	203	1.41	130	10979
840	moddus	4	17	35	234	1.37	132	12357
840	control	1	17	35	196	1.18	132	9285
840	control	2	17	35	201	1.32	137	12022
840	control	3	17	35	206	1.4	131	12397
840	control	4	17	35	244	1.34	133	11432
840	glyphosate	1	17	35	198	1.34	136	11018
840	glyphosate	2	17	35	193	1.31	133	10702
840	glyphosate	3	17	35	216	1.49	131	12178
840	glyphosate	4	17	35	236	1.5	135	12818
226	moddus	1	17	35	226	2.05	132	12695
226	moddus	2	17	35	213	1.55	137	9021
226	moddus	3	17	35	241	1.74	121	7984
226	moddus	4	17	35	269	2.59	121	12018
226	control	1	17	35	231	1.96	120	11934
226	control	2	17	35	236	1.92	130	12708
226	control	3	17	35	267	2.11	124	11474
226	control	4	17	35	274	2.27	129	12539
226	glyphosate	1	17	35	244	2.19	131	14213
226	glyphosate	2	17	35	241	1.76	138	11146
226	glyphosate	3	17	35	239	2.19	138	14503
226	glyphosate	4	17	35	264	2.1	128	12343
283	moddus	1	17	35	218	1.57	150	13043
283	moddus	2	17	35	241	1.73	151	17748
283	moddus	3	17	35	224	1.7	150	14420
283	moddus	4	17	35	211	0.9	141	7583
283	control	1	17	35	239	1.91	147	15849
283	control	2	17	35	236	1.58	145	13923
283	control	3	17	35	234	1.87	141	17662
283	control	4	17	35	269	1.77	137	14758
283	glyphosate	1	17	35	221	1.46	139	12057
283	glyphosate	2	17	35	244	1.59	140	14003
283	glyphosate	3	17	35	231	1.6	143	15491
283	glyphosate	4	17	35	244	1.47	135	10462

299	moddus	1	17	35	259	1.96	116	14295
299	moddus	2	17	35	241	1.48	122	11521
299	moddus	3	17	35	218	1.88	130	16127
299	moddus	4	17	35	257	2.04	125	16763
299	control	1	17	35	264	2.01	115	14949
299	control	2	17	35	244	1.47	125	10955
299	control	3	17	35	241	1.67	121	12800
299	control	4	17	35	267	2.07	123	16236
299	glyphosate	1	17	35	262	1.66	123	12691
299	glyphosate	2	17	35	259	1.67	133	13398
299	glyphosate	3	17	35	226	1.5	122	11202
299	glyphosate	4	17	35	229	1.67	129	13421
613	moddus	1	17	35	216	1.46	130	10025
613	moddus	2	17	35	236	2.4	135	18244
613	moddus	3	17	35	251	1.85	132	12807
613	moddus	4	17	35	231	2.15	135	16702
613	control	1	17	35	234	1.7	128	12394
613	control	2	17	35	246	2.04	132	16790
613	control	3	17	35	259	1.9	128	13658
613	control	4	17	35	267	2.42	130	17749
613	glyphosate	1	17	35	249	2.04	122	13130
613	glyphosate	2	17	35	239	2.02	134	16049
613	glyphosate	3	17	35	241	1.94	130	11681
613	glyphosate	4	17	35	254	2.28	133	17020
540	moddus	1	17	35	218	1.54	123	10108
540	moddus	2	17	35	241	1.93	126	14509
540	moddus	3	17	35	234	2.18	118	14684
540	moddus	4	17	35	239	2.12	120	12796
540	control	1	17	35	203	1.59	115	10095
540	control	2	17	35	234	1.49	126	10966
540	control	3	17	35	224	1.95	113	13614
540	control	4	17	35	259	2.46	113	16043
540	glyphosate	1	17	35	229	1.77	120	11565
540	glyphosate	2	17	35	224	1.73	138	15290
540	glyphosate	3	17	35	234	2.21	123	17030
540	glyphosate	4	17	35	229	1.71	123	13191
838	moddus	1	17	35	193	1.22	136	7976
838	moddus	2	17	35	175	1.25	130	9282
838	moddus	3	17	35	206	1.7	144	12199
838	moddus	4	17	35	224	1.76	132	12836
838	control	1	17	35	218	1.52	134	10597
838	control	2	17	35	188	1.52	124	11654
838	control	3	17	35	198	1.59	128	11731
838	control	4	17	35	206	1.63	131	12141
838	glyphosate	1	17	35	221	1.58	125	10504
838	glyphosate	2	17	35	196	1.47	139	12349
838	glyphosate	3	17	35	218	1.81	143	13766

838	glyphosate	4	17	35	234	2	135	16494
804	moddus	1	17	35	213	1.4	138	11186
804	moddus	2	17	35	213	1.64	137	12700
804	moddus	3	17	35	218	1.58	136	13242
804	moddus	4	17	35	216	1.51	133	14143
804	control	1	17	35	221	1.76	137	14768
804	control	2	17	35	216	1.32	137	11359
804	control	3	17	35	246	1.65	132	13771
804	control	4	17	35	211	1.16	134	8961
804	glyphosate	1	17	35	213	1.49	130	12699
804	glyphosate	2	17	35	206	1.38	132	10289
804	glyphosate	3	17	35	239	1.6	134	14340
804	glyphosate	4	17	35	208	1.41	133	11365
840	moddus	1	16	42	208	1.41	139	11078
840	moddus	2	16	42	183	1.01	141	8190
840	moddus	3	16	42	196	1.01	136	8068
840	moddus	4	16	42	203	1.03	145	8400
840	control	1	16	42	206	1.33	137	10413
840	control	2	16	42	183	0.94	127	6417
840	control	3	16	42	201	1.02	142	8355
840	control	4	16	42	216	1.19	138	9389
840	glyphosate	1	16	42	196	1.07	135	7669
840	glyphosate	2	16	42	170	0.83	147	6507
840	glyphosate	3	16	42	198	1.08	137	8301
840	glyphosate	4	16	42	201	1.13	152	9749
804	moddus	1	16	42	208	1.33	149	11635
804	moddus	2	16	42	191	0.96	141	7347
804	moddus	3	16	42	196	1.16	150	10976
804	moddus	4	16	42	211	1.25	144	10293
804	control	1	16	42	221	1.2	146	8686
804	control	2	16	42	218	1.24	145	8744
804	control	3	16	42	201	1.1	144	8490
804	control	4	16	42	213	1.14	143	9710
804	glyphosate	1	16	42	198	0.99	142	7413
804	glyphosate	2	16	42	185	1.11	149	7361
804	glyphosate	3	16	42	188	1.02	147	8470
804	glyphosate	4	16	42	198	1	144	9182
950	moddus	1	16	42	185	1.55	158	8920
950	moddus	2	16	42	173	1.2	160	8053
950	moddus	3	16	42	180	1.3	160	6684
950	moddus	4	16	42	185	1.09	152	7500
950	control	1	16	42	178	1.3	141	6622
950	control	2	16	42	198	1.27	154	8003
950	control	3	16	42	201	1.55	149	8538
950	control	4	16	42	198	1.37	150	8190
950	glyphosate	1	16	42	191	1.22	161	7766
950	glyphosate	2	16	42	180	1.41	161	9393

950	glyphosate	3	16	42	185	1.38	142	9220
950	glyphosate	4	16	42	175	1.24	163	6696
299	moddus	1	16	42	193	1.03	148	5503
299	moddus	2	16	42	218	1.32	142	10164
299	moddus	3	16	42	193	1.25	143	8144
299	moddus	4	16	42	198	1.05	144	7532
299	control	1	16	42	226	1.24	132	6681
299	control	2	16	42	254	1.54	137	10728
299	control	3	16	42	239	1.49	132	10450
299	control	4	16	42	239	1.25	124	7038
299	glyphosate	1	16	42	188	0.91	151	3564
299	glyphosate	2	16	42	216	1.29	148	8107
299	glyphosate	3	16	42	203	1.4	154	9482
299	glyphosate	4	16	42	208	1.13	148	7388
838	moddus	1	16	42	175	1.08	148	6881
838	moddus	2	16	42	168	0.91	140	6522
838	moddus	3	16	42	206	1.33	151	11338
838	moddus	4	16	42	173	1.2	140	9563
838	control	1	16	42	165	0.89	130	4539
838	control	2	16	42	201	1.19	140	6541
838	control	3	16	42	206	1.4	128	8092
838	control	4	16	42	208	1.45	142	9484
838	glyphosate	1	16	42	160	0.92	140	4471
838	glyphosate	2	16	42	160	0.97	145	6100
838	glyphosate	3	16	42	201	1.01	150	7672
838	glyphosate	4	16	42	206	1.42	148	10620
613	moddus	1	16	42	168	1.39	159	8289
613	moddus	2	16	42	201	1.23	148	8241
613	moddus	3	16	42	208	1.74	154	13994
613	moddus	4	16	42	191	1.5	152	11716
613	control	1	16	42	216	1.63	140	9216
613	control	2	16	42	201	1.58	134	9295
613	control	3	16	42	229	1.82	133	11588
613	control	4	16	42	208	1.53	131	10225
613	glyphosate	1	16	42	173	0.99	157	7069
613	glyphosate	2	16	42	206	1.47	160	10129
613	glyphosate	3	16	42	193	1.35	162	10227
613	glyphosate	4	16	42	213	1.62	159	11293
226	moddus	1	16	42	203	1.86	147	7496
226	moddus	2	16	42	208	1.66	144	8191
226	moddus	3	16	42	206	1.41	128	7322
226	moddus	4	16	42	231	1.84	146	11295
226	control	1	16	42	231	1.74	141	7348
226	control	2	16	42	224	1.76	140	7222
226	control	3	16	42	231	1.84	120	9177
226	control	4	16	42	246	2.29	121	9241
226	glyphosate	1	16	42	208	1.35	150	5878

226	glyphosate	2	16	42	208	1.54	146	8214
226	glyphosate	3	16	42	201	1.3	149	7000
226	glyphosate	4	16	42	196	1.31	159	8590
283	moddus	1	16	42	206	1.26	158	8359
283	moddus	2	16	42	208	1.16	153	8838
283	moddus	3	16	42	196	1.03	154	8055
283	moddus	4	16	42	201	1.22	156	9275
283	control	1	16	42	213	1.26	152	8341
283	control	2	16	42	216	1.12	141	7933
283	control	3	16	42	218	1.24	145	7392
283	control	4	16	42	229	1.49	146	8865
283	glyphosate	1	16	42	216	1.19	150	8448
283	glyphosate	2	16	42	221	1.39	153	10326
283	glyphosate	3	16	42	188	0.77	159	6476
283	glyphosate	4	16	42	213	1.21	149	9106
540	moddus	1	16	42	211	1.31	139	9430
540	moddus	2	16	42	185	1.1	128	5887
540	moddus	3	16	42	201	1.24	142	8810
540	moddus	4	16	42	221	1.36	131	9467
540	control	1	16	42	226	1.34	142	9342
540	control	2	16	42	201	1.26	129	7823
540	control	3	16	42	239	1.75	112	10459
540	control	4	16	42	231	1.36	120	7710
540	glyphosate	1	16	42	196	0.96	140	5616
540	glyphosate	2	16	42	198	1.11	141	5273
540	glyphosate	3	16	42	208	1.37	141	8987
540	glyphosate	4	16	42	188	1.2	141	8619
950	moddus	1	17	42	191	1.76	160	12710
950	moddus	2	17	42	196	1.6	155	11729
950	moddus	3	17	42	201	1.56	139	10766
950	moddus	4	17	42	229	1.96	140	15114
950	control	1	17	42	208	2.11	155	16214
950	control	2	17	42	226	2.11	152	18276
950	control	3	17	42	236	2.09	155	13668
950	control	4	17	42	249	2.22	146	15983
950	glyphosate	1	17	42	191	1.7	158	13655
950	glyphosate	2	17	42	216	1.89	158	12560
950	glyphosate	3	17	42	208	1.46	149	10236
950	glyphosate	4	17	42	226	1.72	145	10856
840	moddus	1	17	42	221	1.42	147	12680
840	moddus	2	17	42	226	1.43	147	12744
840	moddus	3	17	42	226	1.63	145	14080
840	moddus	4	17	42	239	1.49	142	14461
840	control	1	17	42	198	1.51	148	13291
840	control	2	17	42	234	1.51	144	14446
840	control	3	17	42	221	1.57	138	14639
840	control	4	17	42	249	1.32	142	11981

840	glyphosate	1	17	42	191	1.26	144	10955
840	glyphosate	2	17	42	239	1.5	136	12562
840	glyphosate	3	17	42	229	1.6	143	14272
840	glyphosate	4	17	42	244	1.46	145	13335
226	moddus	1	17	42	236	2.13	144	14346
226	moddus	2	17	42	226	1.56	141	9325
226	moddus	3	17	42	262	2.94	143	15964
226	moddus	4	17	42	274	2.76	141	14912
226	control	1	17	42	239	2.19	131	14565
226	control	2	17	42	251	1.97	130	13066
226	control	3	17	42	282	2.89	137	17376
226	control	4	17	42	284	2.38	129	13201
226	glyphosate	1	17	42	249	2.35	144	16776
226	glyphosate	2	17	42	229	1.76	145	11759
226	glyphosate	3	17	42	254	2.22	142	15126
226	glyphosate	4	17	42	254	1.75	135	10847
283	moddus	1	17	42	216	1.54	154	13203
283	moddus	2	17	42	226	1.54	147	15479
283	moddus	3	17	42	226	1.67	149	14116
283	moddus	4	17	42	246	1.39	150	12489
283	control	1	17	42	241	1.76	153	15148
283	control	2	17	42	241	1.65	150	15064
283	control	3	17	42	246	1.85	151	18750
283	control	4	17	42	246	2.04	152	18837
283	glyphosate	1	17	42	244	1.87	150	16647
283	glyphosate	2	17	42	236	1.44	149	13571
283	glyphosate	3	17	42	249	1.98	152	20445
283	glyphosate	4	17	42	264	1.61	147	12473
299	moddus	1	17	42	257	1.85	129	14940
299	moddus	2	17	42	216	1.29	127	10392
299	moddus	3	17	42	251	1.52	125	12557
299	moddus	4	17	42	234	1.62	135	14403
299	control	1	17	42	269	1.66	131	14077
299	control	2	17	42	249	1.42	132	11143
299	control	3	17	42	267	1.77	123	13777
299	control	4	17	42	254	1.5	129	12393
299	glyphosate	1	17	42	254	1.51	129	12118
299	glyphosate	2	17	42	239	1.35	141	11518
299	glyphosate	3	17	42	257	1.83	135	15123
299	glyphosate	4	17	42	229	1.68	146	15294
613	moddus	1	17	42	231	1.62	142	12117
613	moddus	2	17	42	236	2.04	146	16776
613	moddus	3	17	42	231	1.83	145	13963
613	moddus	4	17	42	239	2.24	146	18770
613	control	1	17	42	246	1.75	144	14307
613	control	2	17	42	246	2.21	136	18731
613	control	3	17	42	254	2.12	145	17323

613	control	4	17	42	259	2.26	143	18227
613	glyphosate	1	17	42	249	2.08	140	15321
613	glyphosate	2	17	42	239	1.89	148	16665
613	glyphosate	3	17	42	234	1.89	145	12641
613	glyphosate	4	17	42	246	1.71	138	13258
540	moddus	1	17	42	229	1.43	132	10096
540	moddus	2	17	42	221	1.68	130	12978
540	moddus	3	17	42	254	2.14	124	15058
540	moddus	4	17	42	254	1.75	131	11544
540	control	1	17	42	234	2.04	132	14841
540	control	2	17	42	208	1.38	123	9884
540	control	3	17	42	272	2.35	122	17768
540	control	4	17	42	231	1.93	126	14022
540	glyphosate	1	17	42	216	1.44	140	10965
540	glyphosate	2	17	42	203	1.21	138	10684
540	glyphosate	3	17	42	236	1.67	123	12911
540	glyphosate	4	17	42	236	1.48	127	11793
838	moddus	1	17	42	213	1.41	146	9860
838	moddus	2	17	42	224	1.78	147	14883
838	moddus	3	17	42	208	1.78	145	12888
838	moddus	4	17	42	198	1.17	143	9234
838	control	1	17	42	218	1.34	144	9985
838	control	2	17	42	246	1.74	146	15739
838	control	3	17	42	206	1.75	140	14096
838	control	4	17	42	213	1.41	146	11638
838	glyphosate	1	17	42	216	1.48	129	10193
838	glyphosate	2	17	42	229	1.6	148	14318
838	glyphosate	3	17	42	221	2.1	148	16462
838	glyphosate	4	17	42	203	1.38	148	12500
804	moddus	1	17	42	249	1.75	142	14380
804	moddus	2	17	42	218	1.5	139	11793
804	moddus	3	17	42	239	1.86	141	16125
804	moddus	4	17	42	226	1.39	137	13377
804	control	1	17	42	244	1.9	144	16713
804	control	2	17	42	239	1.57	145	14255
804	control	3	17	42	264	1.72	138	14939
804	control	4	17	42	208	1.28	138	10198
804	glyphosate	1	17	42	239	1.59	140	14503
804	glyphosate	2	17	42	239	1.36	132	10201
804	glyphosate	3	17	42	257	2.11	138	19539
804	glyphosate	4	17	42	218	1.48	142	12771
840	moddus	1	16	49	188	1.02	153	8797
840	moddus	2	16	49	193	0.99	147	8336
840	moddus	3	16	49	191	1.09	151	9671
840	moddus	4	16	49	203	1.1	149	9167
840	control	1	16	49	196	1.04	146	8686
840	control	2	16	49	201	1.27	153	10409

840	control	3	16	49	211	1.13	150	9798
840	control	4	16	49	221	1.05	138	8293
840	glyphosate	1	16	49	188	1.11	138	8143
840	glyphosate	2	16	49	185	0.95	154	7819
840	glyphosate	3	16	49	185	0.98	153	8432
840	glyphosate	4	16	49	198	0.99	147	8281
804	moddus	1	16	49	191	1.09	147	9460
804	moddus	2	16	49	191	1.08	157	9171
804	moddus	3	16	49	206	1.18	153	11356
804	moddus	4	16	49	208	1.32	152	11450
804	control	1	16	49	221	1.2	148	8785
804	control	2	16	49	203	1.18	151	8683
804	control	3	16	49	234	1.48	151	12006
804	control	4	16	49	221	1.08	150	9671
804	glyphosate	1	16	49	211	1.24	149	9758
804	glyphosate	2	16	49	193	0.98	152	6643
804	glyphosate	3	16	49	198	0.99	152	8466
804	glyphosate	4	16	49	213	1.04	148	9841
950	moddus	1	16	49	188	1.19	161	7002
950	moddus	2	16	49	191	1.27	165	8824
950	moddus	3	16	49	183	1.27	156	6358
950	moddus	4	16	49	198	1.38	165	10259
950	control	1	16	49	196	1.41	158	8065
950	control	2	16	49	196	1.23	161	8063
950	control	3	16	49	196	1.2	149	6621
950	control	4	16	49	216	1.55	154	9495
950	glyphosate	1	16	49	178	1.05	162	6728
950	glyphosate	2	16	49	191	1.17	166	8068
950	glyphosate	3	16	49	198	1.28	165	9939
950	glyphosate	4	16	49	173	1.02	151	5098
299	moddus	1	16	49	206	1.15	148	6132
299	moddus	2	16	49	221	1.28	150	10409
299	moddus	3	16	49	203	1.22	154	8556
299	moddus	4	16	49	218	1.36	147	9940
299	control	1	16	49	229	1.49	148	9002
299	control	2	16	49	239	1.14	136	7895
299	control	3	16	49	259	1.63	136	11717
299	control	4	16	49	267	1.8	125	10277
299	glyphosate	1	16	49	198	1.39	162	5825
299	glyphosate	2	16	49	211	1.14	155	7465
299	glyphosate	3	16	49	224	1.55	153	10408
299	glyphosate	4	16	49	208	1.16	158	8111
838	moddus	1	16	49	175	1.08	149	6928
838	moddus	2	16	49	178	1.1	152	8531
838	moddus	3	16	49	191	1.08	154	9408
838	moddus	4	16	49	191	1.18	150	10018
838	control	1	16	49	188	1.21	146	6940

838	control	2	16	49	201	1.26	145	7175
838	control	3	16	49	221	1.63	141	10367
838	control	4	16	49	198	1.28	152	8974
838	glyphosate	1	16	49	191	1.29	148	6599
838	glyphosate	2	16	49	173	1.26	152	8280
838	glyphosate	3	16	49	198	1.39	154	10870
838	glyphosate	4	16	49	193	1.45	154	11247
613	moddus	1	16	49	198	1.44	163	8783
613	moddus	2	16	49	206	1.57	154	10907
613	moddus	3	16	49	198	1.28	146	9765
613	moddus	4	16	49	216	1.68	149	12890
613	control	1	16	49	246	1.78	149	10742
613	control	2	16	49	236	1.66	143	10443
613	control	3	16	49	218	1.57	145	10901
613	control	4	16	49	218	1.41	146	10541
613	glyphosate	1	16	49	183	1.33	164	9914
613	glyphosate	2	16	49	193	1.32	157	8914
613	glyphosate	3	16	49	196	1.53	164	11736
613	glyphosate	4	16	49	193	1.31	171	9846
226	moddus	1	16	49	213	1.46	158	6323
226	moddus	2	16	49	191	1.46	154	7704
226	moddus	3	16	49	201	1.23	143	7119
226	moddus	4	16	49	236	2.14	151	13633
226	control	1	16	49	244	1.79	147	7898
226	control	2	16	49	254	2.3	134	9025
226	control	3	16	49	249	1.81	137	10279
226	control	4	16	49	244	1.73	136	7842
226	glyphosate	1	16	49	201	1.55	156	7004
226	glyphosate	2	16	49	196	1.35	156	7674
226	glyphosate	3	16	49	216	1.45	148	7758
226	glyphosate	4	16	49	203	1.63	154	10322
283	moddus	1	16	49	221	1.39	156	9112
283	moddus	2	16	49	216	1.4	156	10908
283	moddus	3	16	49	206	1.1	156	8693
283	moddus	4	16	49	191	1.13	159	8733
283	control	1	16	49	211	1.42	150	9274
283	control	2	16	49	234	1.56	150	11730
283	control	3	16	49	249	1.45	154	9207
283	control	4	16	49	244	1.62	153	10095
283	glyphosate	1	16	49	193	1.06	160	8043
283	glyphosate	2	16	49	216	1.15	159	8903
283	glyphosate	3	16	49	224	1.16	162	9957
283	glyphosate	4	16	49	224	1.26	159	10090
540	moddus	1	16	49	213	1.39	147	10578
540	moddus	2	16	49	191	0.98	146	5977
540	moddus	3	16	49	218	1.09	141	7657
540	moddus	4	16	49	216	1.37	142	10311

540	control	1	16	49	226	1.39	148	10085
540	control	2	16	49	229	1.51	138	9997
540	control	3	16	49	229	1.34	132	9393
540	control	4	16	49	236	1.31	124	7636
540	glyphosate	1	16	49	185	1.16	148	7171
540	glyphosate	2	16	49	198	1.38	153	7102
540	glyphosate	3	16	49	213	1.46	143	9677
540	glyphosate	4	16	49	211	1.62	139	11440
950	moddus	1	17	49	216	1.87	156	13215
950	moddus	2	17	49	213	1.62	165	12665
950	moddus	3	17	49	231	1.69	154	12909
950	moddus	4	17	49	211	1.54	152	12922
950	control	1	17	49	218	1.88	165	15382
950	control	2	17	49	239	1.92	163	17800
950	control	3	17	49	229	2.25	160	15141
950	control	4	17	49	224	1.96	149	14423
950	glyphosate	1	17	49	211	1.62	162	13284
950	glyphosate	2	17	49	234	2.12	157	14006
950	glyphosate	3	17	49	218	1.28	158	9511
950	glyphosate	4	17	49	208	1.78	146	11277
840	moddus	1	17	49	221	1.43	145	12619
840	moddus	2	17	49	226	1.34	146	11857
840	moddus	3	17	49	239	1.72	149	15279
840	moddus	4	17	49	231	1.56	142	15111
840	control	1	17	49	221	1.52	145	13091
840	control	2	17	49	246	1.57	146	15215
840	control	3	17	49	234	1.5	150	15210
840	control	4	17	49	244	1.27	140	11355
840	glyphosate	1	17	49	234	1.69	146	14923
840	glyphosate	2	17	49	257	1.65	148	15004
840	glyphosate	3	17	49	226	1.49	144	13341
840	glyphosate	4	17	49	234	1.22	146	11276
226	moddus	1	17	49	246	2.38	147	16414
226	moddus	2	17	49	226	2.05	142	12335
226	moddus	3	17	49	269	2.5	145	13713
226	moddus	4	17	49	264	2.24	152	13070
226	control	1	17	49	259	2.31	142	16677
226	control	2	17	49	251	2.24	142	16231
226	control	3	17	49	277	2.36	143	14794
226	control	4	17	49	262	2.27	146	14214
226	glyphosate	1	17	49	254	2.35	152	17747
226	glyphosate	2	17	49	244	2.24	151	15592
226	glyphosate	3	17	49	259	2.58	148	18350
226	glyphosate	4	17	49	249	2.34	151	16274
283	moddus	1	17	49	234	1.52	157	13253
283	moddus	2	17	49	224	1.52	152	15732
283	moddus	3	17	49	244	1.76	151	15011

283	moddus	4	17	49	251	1.62	149	14401
283	control	1	17	49	251	1.9	157	16779
283	control	2	17	49	259	1.65	158	15824
283	control	3	17	49	259	1.67	154	17194
283	control	4	17	49	234	1.43	150	13043
283	glyphosate	1	17	49	257	1.86	158	17483
283	glyphosate	2	17	49	234	1.4	153	13488
283	glyphosate	3	17	49	269	1.66	149	16812
283	glyphosate	4	17	49	236	1.34	148	10475
299	moddus	1	17	49	254	1.47	140	12909
299	moddus	2	17	49	239	1.49	143	13572
299	moddus	3	17	49	267	1.56	144	14908
299	moddus	4	17	49	241	1.55	137	13997
299	control	1	17	49	251	1.46	137	12928
299	control	2	17	49	262	1.61	142	13570
299	control	3	17	49	279	1.73	141	15429
299	control	4	17	49	259	1.98	135	17130
299	glyphosate	1	17	49	246	1.26	143	11163
299	glyphosate	2	17	49	264	1.56	140	13173
299	glyphosate	3	17	49	241	1.22	145	10826
299	glyphosate	4	17	49	216	1.36	148	12534
613	moddus	1	17	49	234	1.94	150	15333
613	moddus	2	17	49	244	1.98	154	17191
613	moddus	3	17	49	239	1.82	146	13963
613	moddus	4	17	49	229	2	148	17036
613	control	1	17	49	267	2.08	148	17561
613	control	2	17	49	274	2.16	135	18271
613	control	3	17	49	262	2.03	147	16847
613	control	4	17	49	239	2.07	146	17007
613	glyphosate	1	17	49	259	1.95	148	15220
613	glyphosate	2	17	49	269	2.31	149	20531
613	glyphosate	3	17	49	226	1.79	154	12746
613	glyphosate	4	17	49	246	2.24	155	19517
540	moddus	1	17	49	224	1.65	137	12123
540	moddus	2	17	49	239	2.04	130	15792
540	moddus	3	17	49	246	1.84	138	14494
540	moddus	4	17	49	229	1.95	136	13348
540	control	1	17	49	231	1.35	142	10608
540	control	2	17	49	241	1.96	131	14994
540	control	3	17	49	239	1.4	128	11076
540	control	4	17	49	239	1.45	135	11333
540	glyphosate	1	17	49	224	1.41	151	11619
540	glyphosate	2	17	49	221	1.75	150	16744
540	glyphosate	3	17	49	241	1.51	140	13289
540	glyphosate	4	17	49	236	1.61	136	13778
838	moddus	1	17	49	198	1	133	6381
838	moddus	2	17	49	226	1.74	146	14420

838	moddus	3	17	49	218	1.69	144	12127
838	moddus	4	17	49	229	1.73	140	13342
838	control	1	17	49	226	1.58	145	11848
838	control	2	17	49	244	1.55	147	14146
838	control	3	17	49	216	1.81	147	15267
838	control	4	17	49	226	1.54	143	12497
838	glyphosate	1	17	49	201	1.43	153	11681
838	glyphosate	2	17	49	229	1.55	147	13702
838	glyphosate	3	17	49	239	1.91	151	15299
838	glyphosate	4	17	49	216	1.51	153	14117
804	moddus	1	17	49	246	1.38	147	11796
804	moddus	2	17	49	226	1.43	134	10810
804	moddus	3	17	49	241	1.51	146	13560
804	moddus	4	17	49	239	1.82	143	18367
804	control	1	17	49	241	1.49	145	13190
804	control	2	17	49	241	1.57	132	13033
804	control	3	17	49	267	1.59	145	14552
804	control	4	17	49	218	1.31	142	10781
804	glyphosate	1	17	49	259	1.77	143	16505
804	glyphosate	2	17	49	244	1.6	146	13206
804	glyphosate	3	17	49	259	1.66	145	16125
804	glyphosate	4	17	49	229	1.44	132	11515
